# Social Insurance for Long-term Care

**DOI:** 10.1007/s12062-022-09366-6

**Published:** 2022-06-01

**Authors:** Maria Karagiannidou, Raphael Wittenberg

**Affiliations:** The London School of Economics and Political Science, London, United Kingdom

## Abstract

The issue of how best to finance long-term care (LTC) is the subject of recent reforms, forthcoming reforms or continuing debate in various countries and remains as relevant and challenging as ever. LTC services are crucial to the wellbeing of large numbers of older adults who need help with everyday tasks.

Demand for LTC for older adults is projected to rise across developed and developing countries as the number of older adults rises. Supply of care services is likely to remain constrained due to shortages of long-term care workforce and financial constraints in many countries, and the financial risks associated with LTC remain.

Financing of LTC is a complicated issue which raises considerations of economic efficiency and incentives, equity including intergenerational equity, the balance of risk between public and private funding, and sustainability of public expenditures.

The aim of this paper is to discuss analytically the case for social insurance as an equitable and efficient way to finance LTC. The paper considers social insurance systems, especially in Germany and Japan, in comparison with safety net tax funded systems such as in England and the USA and more generous tax funded systems such as in Sweden and Denmark. Social insurance has advantages and disadvantages compared with these other systems. It tends to be associated with greater clarity and acceptability since it involves collection of revenues ear marked for LTC and, at least in principle, a link between contributions and benefits on the basis of clear eligibility criteria.

## Introduction

How best to fund long-term care (LTC) for older people is a major social policy challenge for many countries. Demand for LTC for older people is projected to rise across developed and developing countries as the number of older people rises. Supply of care services is likely to remain constrained due to shortages of long-term care workforce and financial constraints in many countries (Colombo et al., 2011; EU, 2021a; Spasova et al., 2018.). The future supply of unpaid care by family and friends, which is the main form of LTC, is uncertain (Colombo et al., 2011; EU, 2021a; Spasova et al., 2018.). The covid-19 pandemic and its impact on LTC has added to the challenges of funding and providing care in the coming years.

LTC services are crucial to the wellbeing of large numbers of people older people who need help with everyday tasks. LTC systems provide a broad range of services assisting older adults in need of care. They enable older people with care needs to live more independently through helping them with activities of daily living (ADLs), for instance, toileting, getting dressed and undressed, bathing or washing, and feeding, and instrumental activities of daily living (IADLs), for example, shopping, meal preparation, and taking medication. The range of services provided varies between countries but generally includes residential care with or without nursing care, day care services, home care services, aids and adaptations, and specialist services at home, including in some countries community nursing and therapy services (Colombo et al., 2011; Joshua, 2017).

The family has a central role in providing support to older people in need of care. The balance between unpaid care and formal services varies between countries, with a greater reliance on unpaid care in (for example) southern European countries than northern European countries, but it is a vital part of care arrangements in all countries (Colombo et al., 2011; Rodrigues, 2015; WHO, 2021). This means that LTC systems need to consider support for unpaid carers as well as for those needing care. In some countries a substantial proportion of LTC is provided by paid carers in an informal grey economy labour market (mainly immigrant women who live in the country legally or illegally). They too are part of the overall LTC system albeit not financed by public funding (Colombo et al., 2011; Spasova et al., 2018; Rodrigues, 2015).

Various classifications have been posited to distinguish different types of LTC systems. The OECD distinguish three models of funding LTC in Europe, that is (i). universal coverage models; (ii). means-tested models and (iii). mixed models (Colombo et al., 2011). The ANCIEN study developed a fourfold classification of LTC use and funding: (i) informal care oriented, low private financing; (ii) generous, accessible and formalised; (iii) informal care oriented, high private financing; (iv) informal care oriented, high private financing (Kraus et al., 2011). In this paper we focus on distinguishing three types of LTC systems: social insurance systems, universal coverage tax-funded systems and safety net tax-funded systems.

Some countries including the Nordic countries have universal tax-based models which provide substantial coverage of LTC needs and costs with only limited charges to service users. Other countries, such as Germany, Netherlands, and Japan, established universal social insurance-based models, which also provide substantial coverage of needs and costs. England and the United States have tax-funded systems with means tests which are often described as safety net systems: they provide coverage for people with low incomes and savings but exclude from eligibility for publicly funded care those with more than relatively modest incomes or savings. Finally, there are countries such as Greece that have adopted a mixture of systems (Colombo et al., 2011; Rodrigues, 2015; Waitzberg et al., 2020).

The issue of how best to finance LTC is the subject of recent reforms, forthcoming reforms and/or continuing debate in various countries and remains as relevant and challenging as ever. It is a complicated issue which raises considerations of economic efficiency and incentives, equity including intergenerational equity, the balance of risk between public and private funding, and sustainability of public expenditures (Wanless et al., 2006). As the population ages, the number of older people with care needs is rising, financial resources are limited, and the financial risks associated with LTC remain. Hence, the funding of LTC, especially in a fair and sustainable way, becomes even more complex and challenging (Waitzberg et al., 2020).

Over the past 20 years a key question on financing LTC systems has been and still is “*who is eligible for what publicly funded care and with what user contributions if any*” (Wanless et al., 2006). A related vital question is the relationship between the formal LTC system and unpaid care: should the availability of unpaid care be taken into account in the eligibility criteria for assessing need for publicly funded care and should unpaid carers themselves be eligible for LTC benefits or services?

An important feature of LTC is the opportunity cost of unpaid care by family and friends. Although these costs do not involve a monetary transaction, they can be substantial especially if the carer needs to reduce their hours of work or give up work completely to provide intensive care. For example, it has been estimated that in England almost 42% of all care costs for people with dementia relate to unpaid care (Spasova et al., 2018; Wittenberg et al., 2019).

One of the principal questions in the design of any LTC system is the description of needs and the definition of eligibility/entitlement for LTC benefits and services. Assessment and eligibility instruments and algorithms influence the overall efficiency and equity of the system. It is one of the most important aspects for steering LTC systems in terms of: regulating the number of beneficiaries and, by doing so, having a baseline for calculating costs and resources necessary to run the system. It thus has an impact on the overall effectiveness of the system, promoting clarity and transparency and promoting equity (Eleftheriades & Wittenberg, 2013; Fernández et al., 2009; Fernández & Forder, 2010).

There has also been debate over at least the last decade about improving access to LTC and the quality of LTC services. This requires further action for most developed countries to secure a sustainable and effective funding mechanism for their social protection systems and consequently for strengthening the provision of a fair and economically efficient LTC system. It has been crucial for most countries to find ways to cope with these challenges. As a consequence, most EU countries, England and some Asian countries have been reforming or planning to reform their LTC services with emphasis on eligibility criteria and financing schemes (Joshua, 2017; Spasova et al., 2018).

The aim of this paper is to discuss the case for social insurance as an equitable and efficient way to finance long-term care. The paper takes a broad comparative approach and discusses social insurance systems, especially in Germany and Japan, in contrast to safety net tax funded systems such as in England and the USA and more generous universal coverage tax funded systems such as in Sweden. It presents the case for social insurance as an effective way to finance long-term care in the context of rising demand and constrained resources. It considers both its strengths relative to other financing systems and its limitations. It is innovative in presenting a recent broad comparative overview of the case for social insurance.

The question of how best to finance long-term care is clearly far from the only policy issue of concern in the field of care for older people. There are important issues of what forms of care to provide, whether to provide cash or services, how to support unpaid care, whether services should be provided by the public sector or the independent sector, how to promote quality of care and what should be the role of government in regulating the care sector. This paper however focuses on the issue of financing long-term care. This topic seems relevant for the ongoing debate in England and also for continuing debate in other countries. While the reform proposals which the UK government propose to implement in England do not amount to a move to social insurance, they involve a move toward partial hypothecation of specific sources of funding for LTC; and, as discussed below, hypothecation is an important feature of social insurance systems compared with tax funded systems.

## Demographic trends

Europe is ageing much faster than in the past. According to the latest Eurostat data, in 2020, 20.3% of the European Union (EU) population was aged 65 years or over (Eurostat, 2020). Across the EU Member States, the highest share of older people in the total population in 2019 was observed in Italy (22.8%), followed by Greece (22.0%), Portugal and Finland (21.8% each), Germany (21.5%) and Bulgaria (21.3%) (EU, 2021b).

According to the latest EU data, the number of people potentially in need of long-term care services is expected to rise significantly, that is from 30.8 million in 2019 to 38.1 million in 2050. In addition, the prevalence of activity of daily living limitations increases significantly with age and gender: women and older adults have higher risks of developing a degree of disability that will require long-term care assistance. As a result, LTC costs are projected to increase greatly over the coming decades from 1.7% of GDP in 2019 to 2.5% of GDP in 2050 (EU, 2021a).

The working-age population (people aged 18–64) is projected to shrink by 18% by 2070, to approximately 220 million from 265 million in 2019. This is expected to have a negative impact on economic growth and consequently on countries’ ability to fund social protection policies, including LTC services. According to recent Eurostat data, 18.5% of all people aged 65 and older in the EU were at risk of poverty or social exclusion in 2019 and the absolute number of people at risk of poverty is likely to increase in the future. Moreover, the number of people that depend on long-term care (LTC) services is projected to increase by 23.5% by 2050 (EU, 2021b). It also worth noting that LTC needs increase substantially with age. In addition, women, people living alone, people with lower socioeconomic status and in poorer health have higher a risk of needing LTC services (EU, 2021a; EU, 2021b).

These demographic changes will impact countries’ ability to offer a sustainable and effective framework of social protection, including long-term care for older people. The rapid growth of the older population, as a result of increasing longevity and ageing of the “baby boomers” generation, will lead to a significant increase in the number of older people with substantial care needs. This, in turn, will require expanding and strengthening long-term care and will increase the financial pressure on LTC systems and the wider welfare state (Joshua, 2017; Spasova et al., 2018).

## Insurance for long-term care

There is wide variation in the needs for and costs of LTC required in the later years of life. Some people do not require any formal LTC services, either because they do not experience need for help with personal care or domestic tasks even toward the end of life or because their needs are met by family members. Others have substantial need for LTC services for several years, for example if they have dementia, survive to the severe stage of the condition, enter a care home and survive there for several years (Colombo et al., 2011; Joshua, 2017; Spasova et al., 2018).

Forder and Fernández (2009) estimated that, while 25% of older people have zero lifetime costs of LTC services, 10% have costs exceeding £100,000 during the final years of life. Thus, the cost of LTC can become very substantial. While the estimated average lifetime cost of LTC in England was 21,400 GBP (Forder & Fernández, 2009), costs can be much higher, especially for people needing residential or intensive community-based care for more than a short duration (Forder & Fernández, 2009).

Since need for LTC in the later years of life is a risk that everyone faces, a flourishing market for private long-term care insurance might be expected. People who are aware of the substantial risk of needing LTC toward the end of life and who are risk averse have a substantial incentive to purchase LTC insurance if it is available and affordable (Barr, 2010). A risk averse person who is aware that they might potentially require residential care during the last 3 or 4 years of their life at a total cost in excess of £100,000, may prefer to pay a premium of, for illustrative example, some £20,000 or £30,000 or more with certainty to avoid this risk. It is in principle more efficient for people to have the option to purchase such insurance rather than seek to save £100,000 in case they should need costly LTC over an extended period (Barr, 2010).

A crucial advantage of insurance is its efficiency in the face of risk aversion. As Barr (2010) argues, ‘self-finance (i.e. financing long-term care out of personal savings or a long-term care savings account) is an inferior solution. Where someone is risk-averse the possibility of pooling risk is welfare-enhancing’. It is important to note that this efficiency gain arises in principle from any form of risk-pooling through insurance, including private insurance, social insurance and tax-based systems which also involve risk pooling. The extent of risk pooling is greater under relatively generous tax funded systems such as in Sweden than under safety-net systems such as in England with its tight means-test. This efficiency argument is the key reason which the Commission for the Funding of Social Care (CFSC, 2011) adduced to advocate the introduction of a lifetime cap on liability to meet care costs in England. The objective was to ensure that the risk of high life-time costs would be pooled even for people with substantial incomes and savings.

In practice private LTC insurance designed to fund the full costs of care faces substantial market failures (Barr, 2010; Comas-Herrera et al., 2010). On the supply side, there is considerable uncertainty about future levels of need for LTC and future LTC costs. Private insurance can handle risk where the distribution of the adverse event, such as mortality rates by age and gender, is known. It cannot handle uncertainty where the distribution of the adverse event is unknown (Eling & Ghavibazoo, 2019).

The challenges facing private LTC insurance, including its lack of availability in some countries, limitations in other countries and high cost, constitute a strong case for public funding of LTC. Public funding pools the risk of high LTC costs over the widest possible group, the entire population. It also enables the resources for LTC to be raised in a manner that is based on a person’s income rather than on their actuarially assessed risk. Public funding meets at least three of the four aims of the welfare state which Hills (1997) described, which are:

- insurance of all against risks such as illness or unemployment.
- redistribution towards those with greater needs, such as for health care, disability or family circumstances,
- smoothing out the level of income over the life cycle, and.
- stepping in where the family ‘fails’.

It involves insurance against the risk of high lifetime care costs, sometimes described as ‘catastrophic’ costs. It redistributes resources from those with lower care needs who receive little or no LTC to those with higher care needs who receive substantial LTC. It also redistributes resources across the life cycle, since LTC for older people is concentrated in the last years of life but is funded by social insurance contributions or taxes paid during all or much of people’s working lives. It could also be regarded as offering support where the family is not able, or no longer able, to provide the support which the person with care needs requires (Barr, 2010; Eling & Ghavibazoo, 2019).

## Publicly funded LTC systems

Public funding of LTC raises a series of issues about the ways in which resources are raised to finance care and the eligibility criteria for determining who should receive LTC benefits and whether they should make any contribution to the costs of their care (Eleftheriades & Wittenberg, 2013). As indicated above, we consider three types of LTC systems: social insurance systems, universal coverage tax-funded systems and safety net tax-funded systems. We first discuss what distinguishes social insurance and tax-funded systems.

Social insurance systems are financed out of social contributions, generally from employees and employers. Those funds are committed to the particular purpose for which they have been raised. The LTC system is based, at least in theory, on the insurance principle, with entitlement following contribution. The contributory principle may be considered to distinguishes social insurance from other financing systems, but in practice the distinction between social insurance and tax-based systems is more nuanced (Barr, 2010).

In tax-funded systems the costs of LTC are met out of the general revenues generated by taxes levied by central or local government. This might include revenues from a range of taxes such as income taxes (direct taxes) and taxes on specific goods and services (indirect taxes). Governments decide what proportion of these revenues should be spent on each public service. There is no fixed rule about what proportion will be allocated to LTC (Barr, 2010; Colombo et al., 2011; Joshua, 2017; Wittenberg et al., 2002).

Social insurance implies a scheme in which resources are raised for specific services or benefits, and are ear-marked, or hypothesised, for those services. The income from social insurance contributions for LTC in Germany and Japan are hypothesised for LTC and cannot be transferred to fund other services. The income from these contributions can however be supplemented, where necessary, with contributions from general tax revenues as is the case in Japan. It is also possible for revenues from a source treated as a tax to be ear-marked for LTC. In England local authorities can raise an addition to their local council tax that is specifically for adult social care. Nevertheless, hypothecation of revenues is in general an important difference between social insurance and tax-funded systems (Barr, 2010; Colombo et al., 2011; Joshua, 2017; Wittenberg et al., 2002).

Social insurance may suggest that that there is a link between contributions and benefits, for example that contributions need to be paid for a number of years to qualify for benefits or that benefit levels depend on contribution records. This is not, however, the case for LTC social insurance. In Germany almost all workers and pensioners are required to pay contributions and are eligible for benefits which depend on need and not on past contributions. While wealthier people can opt for private insurance instead of social insurance, the private insurance policy must offer benefits as good as the social insurance scheme (Roland et al., 2021).

The obligation to pay social insurance contributions ear-marked for LTC seems likely to lead to an expectation that there will be clear rules for eligibility to receive LTC benefits. The public may reasonably expect that, even if there is no link between the level of their contributions and the level of the benefits which they could receive if they develop care needs, they will have a right to benefits on clearly stated conditions. Germany’s eligibility criteria for receipt of LTC payments or services are more specific than the minimum national eligibility criteria in England. This point is discussed further below. While eligibility criteria need not differ between social insurance and tax-based systems, in systems where people pay earmarked social insurance contributions, they seem likely to expect formal entitlements if they meet specified criteria (Eleftheriades & Wittenberg, 2013; Dyer et al., 2019; Joshua, 2017).

Social insurance contributions generally take the form of payroll taxes. In Germany the contributions are currently 3.05% of earnings for those who have children and 3.3% for those without children, shared equally between employees and employers (EU, 2021a). In Japan the contributions are also based on earnings and are levied on people aged 40 and over (Ikegami, 2019). In countries with tax-based LTC systems, however, resources for publicly funded LTC are drawn from general taxation, in the case of England from a combination of central and local taxes.

Social insurance does not necessarily mean that there are no user charges for those receiving services or benefits. In Germany there are no user charges, but benefit rates are not necessarily sufficient to fund the full care package the person requires, especially in the case of residential care where they do not usually cover the full care home fee. In Japan, however, a co-payment is required, usually 10% of the cost of the care (EU, 2021a; Ikegami, 2019). While social insurance is not inconsistent with user charges, it would seem improbable that a social insurance scheme could completely exclude from benefits people with care needs on the grounds of their incomes or savings (Rothgang, 2010; Wittenberg et al., 2002). It is unlikely to be compatible in practice with a ‘safety-net’ system such as the current system in England.

## Funding systems in different countries

### Social insurance

There are considerable differences between the countries that finance their LTC system through social insurance and differences between those that have tax-based systems. Social insurance systems differ in terms of who contributes how much at different stages of their lives and who is entitled to how much LTC benefit under what eligibility criteria. For instance, In Japan, access to LTC scheme is offered only to people aged 65 and over, while in Germany all individuals, irrespective of their age, are entitled to LTC insurance benefits (Ikegami, 2019; Rothgang, 2010). We consider the differences in the social insurance systems in these two countries.

#### *Germany*:

The German LTC social insurance system is based on Bismarck’s values and parallels Germany’s health insurance system. It has been widely regarded as an exemplar of the social insurance model in the European and international context and has been mentioned in the longstanding debate in England about reforming LTC. What underlies the German system is that individuals pay contributions which are mandatory and have a legal entitlement to certain levels of benefits if they meet the eligibility criteria. These criteria are laid down in greater detail than England’s national minimum eligibility criteria (Dyer et al., 2019; Eleftheriades & Wittenberg, 2013; Gerlinger, 2018; Hashiguchi & Llena-Nozal, 2020). There are no user charges, and no means test in the German system. It marks a considerable contrast (social insurance, entitlement, no formal means test) to the English system (Joshua, 2017). The German system experienced important reforms in recent years. One key aspect was the widening of the eligibility criteria. In addition, in recent years, the number of people receiving care at home is rising, while the number of people living in care homes remains unchanged (EU, 2021a).

Functional disability in terms of capacity to perform ADLs, IADLs, and cognitive impairment are all taken into account in the German system of eligibility requirements. If a person is unable to perform routine activities of daily life for the last six months in the areas of personal hygiene, nutrition, or mobility, because of physical or mental illness/disability, she or he may be eligible for long-term care benefits. (Eleftheriades & Wittenberg, 2013; Gerlinger, 2018). The LTC system offers three main types of benefits for those entitled, that is care allowances, home care and residential care. However, if benefits are taken as cash, the value of the benefit is considerably lower than if taken as a care package (Eleftheriades & Wittenberg, 2013). In addition, people in need of care can receive a number of additional services, including respite care, part-time individual day and night care, nursing aids (for instance, special beds), and care management. Moreover, there are generous benefits for carers aiming to balance care and work demands. They include a guaranteed right of return to full time work after a temporary period of part-time work due to caring responsibilities and an entitlement to reduced weekly working hours for at least two years (EU, 2021a).

Although the LTC benefits cover most of the costs of services, they do not necessarily cover the full costs. The most recent available data (2017) indicates that 21.4% of total LTC expenditure was covered privately. Thus, in 2019 the German government adopted a new provision to reduce private payments by families caring for their older relatives. The new legislation introduced a threshold of 100,000 euros, such that only children of people needing care with individual income above 100,000 euros annually will be required to cover any additional LTC costs (EU, 2021a).

There are concerns about the future financial sustainability of the German system in view of population ageing. The contribution rate was held constant at 1.7% for many years following the introduction of the social insurance system in 1995 but has been increased in recent years, to 3.05% in 2020. There has been an increase in public expenditure on LTC services to 35.54 billion euros, mainly due to the 2017 reforms including the widening of eligibility criteria (EU, 2021a).

#### *Japan*

Japan implemented a public long-term care insurance (LTCI) system in 2000. The Japanese LTCI is a mandatory contributory scheme under which half the funding is from social insurance contributions paid by people aged 40 and over and half is from revenues from general taxation (of which 50% are from national government, 25% from the prefectural government and 25% from the municipalities). The contribution rate is determined by the amount of income required to fund LTC services for those who meet the eligibility criteria. All LTC services are subject to co-payments (Colombo et al., 2011; Ikegami, 2019). These have been set at 10% of the costs of care. However, a reform of the Japanese system in 2014 led to an increase in the contribution rate of people with higher incomes or pensions to 20%, and a further reform in 2018 introduced a 30% co-payment rate for those with very high incomes (Roland et al., 2021).

All people aged 65 years or older have access to the LTC system in Japan under the LTCI system regardless of their income or availability of family support. People aged between 40 and 64 years can also access LTC services if they have been assessed as eligible due to a disease or disability. Benefits take the form of services: there is no option to receive cash benefits instead of services (Colombo et al., 2011; Ikegami, 2019; McGrattan et al., 2018).

Long-term care benefits, including institutional, home and community-based services, are accessed via the care manager. The role of the care manager is central to the Japanese LTC insurance system. Carer managers are responsible for navigating people into services and benefits, and they also offer screening as well as counselling and support where needed (HiraKawa, 2016; Ikegami, 2019).

Eligibility for receipt of LTC services is assessed using a standardized questionnaire on activities of daily living. Benefits are set by seven eligibility levels. The results of the assessment are reviewed by a local committee that determines the level of need and appropriate services. Similarly, to Germany, each level of need has its own service range and limit in terms of duration and amount. If the family or the individual requires more services than offered under the assessed level of need, they meet most of the costs. However, low income individuals contribute less. Needs are reassessed every two years or upon request following a change in the person’s condition (Ikegami, 2019; McGrattan et al., 2018).

### Universal tax funded schemes

Nordic countries (Sweden, Norway, Denmark, Finland) provide typical examples of universal, tax funded LTC systems. Under these countries’ schemes, LTC is funded by municipal taxes and government grants. LTC coverage is provided through a single programme which is part of the wider welfare and health-care system. One of the core characteristics of the Nordic LTC system is decentralisation, that is municipalities and local governments have extended autonomy in providing LTC services. As a result, there is local variation in eligibility criteria and services provided. However, central government has the main responsibility for setting the policy objectives for care of older adults. However, Finland has recently undergone a reform aiming to centralise its health and social care system in order to improve access to services, co-ordination and prevention. The 170 primary healthcare authorities were centralised to only 20 joint health and social care units (Polin et al., 2021). In contrast, Denmark’s government plan focuses on expanding decentralisation of health and social care services (Polin et al., 2012).

Nordic countries have some of the largest shares of GDP spending on LTC in Europe (Denmark 3,6%; Norway 3,5%; Sweden 3,5% and Finland 2,0–2,5%) (Barber et al., 2021; EU, 2021a). In addition, under the Nordic LTC framework, emphasis is placed on healthy ageing interventions and, overall, on policies aiming to support older people to remain in their home as long as possible. Examples of services provided are personal care in institutions or at home, home adaptations, nursing homes, assistive devices (Barber et al., 2021; Colombo et al., 2011; EU, 2021a).

With its universal, tax-based system Sweden may be considered a model of universal tax funded care for older adults. Overall, health and social care services for older people are a fundamental part of the Swedish welfare state. Currently, LTC funding consist of 90% local municipal taxes and 5% national government grant. The remaining 4–5% of total LTC costs is covered by out-of-pocket payments, which is the lowest proportion met by out-of-pocket payments among the EU-27 countries. Coverage is generous, as stated by the Social Services Act. All citizens in need of care are potentially entitled to access social care services (Barber et al., 2021; Colombo et al., 2011; EU, 2021a). Access to LTC is based on needs-assessment only, with eligibility criteria determined locally, as there is no national framework of eligibility criteria. Each municipality is free to decide on assessment tools and processes, services provided, and levels of services offered. Assessment is conducted annually, although there are some exceptions. In-kind benefits (vouchers for care, home and institutional care) are prioritised over cash-benefits, which are mainly for carers and vary across municipalities (Barber et al., 2021; Colombo et al., 2011; EU, 2021a).

An important feature of the Swedish LTC system in recent years is the introduction of incentives for municipalities and local governments to strengthen home-based services for older people versus institutional care. Thus, only the most dependent older adults can currently access institutional care in Sweden. This policy has led Sweden to have the largest decrease between 2007 and 2017 in LTC beds among OECD countries - a reduction of 15 beds per 1000 people over 65 years old compared with an average 3.4 beds reduction in OECD countries generally. It is notable that the recent restrictions in LTC coverage resulted in increases in both unpaid care by the family and use of privately purchased care (Barber et al., 2021; Colombo et al., 2011; EU, 2021a).

Another typical example of universal, tax-based systems is Denmark, which shares a lot of common characteristics with Sweden, including high level of decentralisation reflected in the central role of municipalities, in terms of financing, structuring and running LTC services, a more general national legislative framework, and local based eligibility criteria. In addition, the Danish system covers all citizens in need of care independently of their age, income, assets or the availability of unpaid care. LTC related out-of-pocket payments accounted for only 0.2% of GDP in Denmark in 2017, the lowest proportion in the EU-27 (Barber et al., 2021; Colombo et al., 2011; EU, 2021a). The LTC sector in Denmark provides four types of services: prevention, rehabilitation, home care and institutional care. Denmark has over the last two decades actively promoted community-based care and preventive interventions rather than care homes. By prioritising preventive policies and home care, the Danish LTC system aims to reduce the fiscal pressure on the older adult care system. Therefore, the system focuses on interventions aiming to increase older people’s ability to stay as long as possible and safely in their own home and community and to delay or avoid institutionalisation. As a result, coverage has decreased in recent years through stricter assessment criteria as the criteria for accessing residential care became tighter (Barber et al., 2021; Colombo et al., 2011; EU, 2021a).

### Safety net tax funded (means-tested) schemes

Means-tested schemes provide a social safety net with targeted eligibility for persons with a low income and high level of need. Under such funding schemes, LTC is funded from general tax revenues. Eligibility for publicly funded care is subject to an assessment of care needs and an assessment of the person’s financial circumstances. Income and/or asset tests are used to determine eligibility for publicly funded LTC care. Each country determines specific limits or thresholds, and only those individuals falling below that set of thresholds are entitled to publicly funded LTC services or benefits. Those who are so entitled may then be required to contribute to the costs of their care. Publicly funded LTC is thus prioritised to those with the highest care needs and with less income and assets. The principle aim of the means-test is to protect those individuals who would otherwise be unable to pay for LTC themselves and would have unmet needs for care (Barber et al., 2021; Colombo et al., 2011; Joshua, 2017).

#### *England*

The English system is a safety-net social care system within a Beveridge welfare state and is funded from general taxation and income from user charges. Local authorities in England are responsible for assessing needs for social care of adults living in their area, setting eligibility criteria for publicly funded care and commissioning services to meet needs. There are national guidelines for many of local authorities’ responsibilities, including national minimum eligibility criteria, a national means-test for residential care and guidelines on the means test for community-based care (Comas-Herrera et al., 2010; Wittenberg & Malley, 2007). The Care Act 2014 and associated regulations and guidance introduced a range of changes to adult social care in England (Department of Health and Social Care, n.d.), including national minimum eligibility criteria for publicly funded LTC, increased entitlements for unpaid carers and new responsibilities for local authorities in respect of managing care markets. The Care Act also contains provisions to reform the financing system, but these were not brought into effect in 2016 as originally planned.

After substantial delay in September 2021 the UK government announced a new reform of the social care system in England and a plan to fund this reform. Currently, anyone with assets over £23,250 has to pay for their care costs in full, subject only to an NHS contribution to the costs of nursing care in care homes. The value of the person’s home is taken into account as part of their assets if they enter a care home and their former home is not occupied by a spouse or other relative (HMG, 2021).Under the reform proposal this threshold will rise from £23,250 to £100,000 such that people with less that £100,000 of assets will be able to access the social care system, but they will still be expected to contribute to the costs of their care from their assets (above £20,000) and their incomes. There will also be a new lifetime cap of £86,000 on liability to meet care costs. The new plan will be funded by a rise in national insurance contributions of 1.25% point for employees and 1.25% points for employers (HMG, 2021).

#### *USA*

In USA there is a social safety net system for financing and providing LTC services targeted to people with limited ability to afford the costs of their care. The main public programme funding LTC is Medicaid. It provides health care coverage, including LTC, to millions of Americans who have low incomes. It is administered by the states according to federal requirements and is funded jointly by the states and the federal government. Publicly funded LTC services in the USA are therefore funded from general tax revenues and through co-payments determined by assessment of needs and means-testing (income and assets) (Barber et al., 2021; Weiner et al., 2020).

In addition, in limited cases Medicare acts as a public payer of LTC services for people over 65 years old. Although Medicare is not considered a major public payer for LTC, the U.S. Congressional Budget Office includes some Medicare post-acute benefits (for instance, skilled nursing and home health service) as part of LTC spending (Barber et al., 2021).

In 1997, the Programme of All-Inclusive Care for the Elderly (PACE) (or in some cases named as Living Independence for the Elderly programme-LIFE) was introduced as part of the Medicare and Medicaid programmes. The aim of the PACE programme is to enable older people eligible for nursing home care to stay in the community, rather than in a nursing home, as long as possible. PACE offers a range of home and community services, including but not limited to in-home personal care assistance and adult day care. However, PACE is not a national programme: currently, PACE is available in 30 States To be eligible for the programme, people have to be at least 55 years old and be eligible for Medicare and Medicaid. Thus, people have to fulfil needs assessment criteria based on ADLs, IADLs and cognitive impairment and have low income and assets (Barber et al., 2021; USA Government, 2021).

In recent decades the USA’s LTC system has gradually shifted towards community-based care (Wiener et al., 2018). Recently the Biden Administration proposed a bill before Congress under which Medicaid would expand its coverage and community services (mainly home-based care) would be increased. Over the next eight years, the US government will spend $400 billion mostly for home and community care by expanding the coverage of Medicaid to such services. Currently, home-based services are not available by the states, but under the new law the states will provide community and home care services (White House, 2021a and 2021b). (.

According to a recent study, 54% of middle-income older adults in the USA will not be able to afford their out-of-pocket costs for their care needs in 2029 (Pearson et al., 2019). This suggests that there is a need for new LTC policies in the USA to meet the increasing demand for affordable LTC services.

### Mixed systems

Some countries have adopted a mix of tax-funded, social insurance and safety net approaches, for example, Australia, Austria, Canada, France, Greece, Ireland, Italy, and Spain (Feng & Glinskaya, 2020). One common characteristic of these countries is that they do not have a single payer for their LTC system, but they use a mix of funding schemes in different combinations. They have different programmes for different LTC services and/or cash benefits with different eligibility criteria. Some of them have established LTC systems and others only fragmented services. It is, therefore, difficult to group these countries according to the structure and organisation of their services as they vary significantly (Colombo et al., 2011; Feng & Glinskaya, 2020).

Greece is an example of a mixed system in terms of funding LTC, since LTC services are mostly (79%) funded by the social insurance system (compulsory contribution for health and social care), partly (18%) by general taxation, and 4% by non-profit institutions (EU, 2021a). In addition, for some services priority is given to people with very low incomes and to those living alone; but there is no official means testing process. Overall, Greece has a fragmented system of publicly available LTC services for older adults, with very limited home and community-based services, with very low public spending on LTC (0.2% as share of GDP) and high budgetary restrictions, leading a significant share of LTC to be financed by out-of-pocket payments (Spasova et al., 2018). There is limited access to publicly funded LTC through very tight eligibility criteria (Spasova et al., 2018). As a result, the family plays a central role in LTC supply, and for those without close family or friends it may be difficult to get the care needed. Overall, the services provided are of limited coverage, and their supply falls well short of demand (Ziomas et al., 2018).

## Criteria for assessing LTC funding systems

The Wanless report (2006) on adult social care in England sets out six criteria for assessing LTC systems: fairness; economic efficiency; choice; physical resource development; clarity; sustainability/acceptability (Wanless et al., 2006). It is interesting and informative to consider whether social insurance systems or tax-based systems are more likely to meet these criteria. Much depends on aspects of countries’ LTC arrangements other than whether they have social insurance or tax-based systems, but some aspects of their arrangements are associated with which of these systems countries have adopted.

Pooling risks through insurance is substantially more efficient than leaving each person to meet their own LTC costs from their own resources. Since private LTC insurance is subject to market failure, this requires public insurance. While this might suggest social insurance, it is important to note that it is not only social insurance schemes that pool risks: tax-based schemes also pool risks across the population. The difference between the systems does not lie in whether or not risks are pooled but in the way in which revenues are raised and allocated to fund LTC, as discussed above (Comas et al., 2010; Eling & Ghavibazoo, 2019; Roland et al., 2021; Wittenberg et al., 2002).

While there are different ways of defining fairness, a widely adopted approach is equal resource for equal need. While achieving this requires a public scheme, that scheme could in principle be either a social insurance or a tax-based arrangement. The key issue is how liability to meet the costs and eligibility to receive benefits are defined. Social insurance has the potential to be fairer in terms of how resources are raised. The contributions are based on earnings, which mean that they are progressive, with richer people contributing a higher proportion of their income than poorer people, so long as there is no upper earnings limit on liability to pay contributions. Taxes revenues are generally raised through a combination of direct taxes such as incomes taxes and indirect taxes such as value added taxes (VAT) on purchase of goods and services. While the former are progressive, the latter tend to be regressive with poorer people paying a higher proportion of their incomes on indirect taxes than richer people (Comas et al., 2010; Roland et al., 2021; Wittenberg et al., 2002).

Social insurance systems may in some respects offer greater clarity than tax-based systems. This is mainly because countries with social insurance systems may need to have eligibility criteria that are more detailed and specific than countries with tax-based systems. If contributions are levied to fund a specific service, the public who are paying those contributions ear-marked for a specific purpose can reasonably expect that their contributions guarantee an entitlement to benefits on clearly stated eligibility criteria in a manner analogous to private insurance (Barr, 2010; Roland et al., 2021; Wittenberg et al., 2002). A significant disadvantage of tax-funded systems relative to social insurance is less transparency about the relationship between revenues raised and LTC funding (Barber et al., 2021). According to Rothgang and Engelke (2009), this may have a negative impact on people’s willingness to pay higher taxes to fund LTC.

The criterion for which there is arguably the greatest difference between social insurance and tax-based systems is sustainability/acceptability. Tax-based systems may offer greater flexibility and adaptability in flexing the resources allocated to LTC depending on changes in LTC needs over time. The revenues raised through social insurance depend on the state of the labour market unless and until contribution rates can be changed, which can be politically challenging (EU, 2021c; Barber et al., 2021). In tax-based systems, the broader tax base renders the level of resources available for LTC less closely influenced by the state of the labour market and the share of wages in GDP (EU, 2021c and 2021d).

Social insurance ensures that at least the revenues raised from the contributions are hypothecated for LTC and cannot be transferred to other services. Under a tax-based system, in the absence of ear-marked contributions, there can be high competition with other areas of public spending which may have broader political support, for instance, education, health care or pensions. This may lead to difficulty increasing the funding for LTC in line with increases in demand. In countries facing economic difficulties or crises, there could be no increase even a decrease in allocation of revenues to LTC (EU, 2021c and 2021d; Barber et al., 2021).

Means-tested tax-based systems raise some further issues. They offer a safety net framework to those individuals who do not have the means and resources to meet the costs of their care. Public funding for care is targeted to people with low incomes and savings, who have priority under this system. Undoubtedly, this approach is effective in limiting LTC costs, even in the cases where the cost per eligible beneficiary is high, by minimising the eligible population to low income groups (Colombo et al., 2011; Klimaviciute & Pestieau, 2018).

Safety-net funding frameworks may result in increasing unmet needs if the means test threshold creates a group of people who are not poor enough to be eligible but who nevertheless struggle with care expenses (Fernandez, et al., 2009; Feng & Glinskaya, 2020; Weiner et al., 2020). They tend to create large gaps in coverage for the vast majority of the middle-class population who face a risk of high lifetime care costs but cannot insure against them because of lack of adequate, or any, private insurance (Feng & Glinskaya, 2020; Klimaviciute & Pestieau, 2018).

In countries which have universal coverage health-care systems but means-tested LTC systems, such as England, it is likely that there will be inequalities and perverse incentives with unmet need for LTC leading to potentially avoidable use of health care services (Colombo et al., 2011; Feng & Glinskaya, 2020). In addition, the high administrative costs of running means-testing processes cannot be ignored (Colombo et al., 2011). Overall, means-tested schemes for accessing LTC services can lead to increasing unmet needs which can lead to families facing high LTC expenditure (Fernandez et al., 2009).

## Conclusions

The funding of LTC is a complex and potentially controversial but very important policy issue. LTC is vital for the wellbeing of many older adults as well as many younger adults with physical, learning or mental health disabilities. In the light of new challenges to countries’ ability to fund social protection, arising from population ageing, shrinking workforce and transformation of the labour market, there is an urgent need to ensure that LTC funding systems are sustainable. In parallel, there is a need to maintain, and preferably improve, the coverage of LTC systems and the provision of high-quality services (EU, 2021d; Joshua, 2017; Rodrigues).

The difference between social insurance and tax-based systems does not clearly lie in one specific issue but in a combination of issues. Social insurance implies a link between contributions and benefits, but in practice there may not be a direct link between the level of contributions paid and benefit entitlements. There may however be an expectation of entitlement to benefits in return for contributions if clearly specified eligibility criteria are met. Social insurance schemes tend to be funded through payroll taxes with their revenue hypothecated for specific services. While they may involve co-payments, they tend not to involve means tests which preclude people with substantial incomes or savings from benefits (Barr, 2010; Wittenberg et al., 2002).

The capacity to fund welfare state services including LTC in the future will depend on future economic growth, which will reflect changes in the size of the workforce and in productivity. This holds for social insurance and tax-based systems. The latter however have a wider base than the former. Social insurance schemes are generally funded through payroll taxes levied on employees and employers, and the revenues raised are hypothecated for specific services, in this case LTC. This renders the revenue source sensitive to changes in the labour market including variations in employment rates and average earnings. Under tax-based systems revenues from specific taxes are not normally hypothecated for specific services. Since governments can decide what proportion of the revenues to allocate to each service, resources for LTC are less vulnerable than under social insurance to fluctuations in the labour market (Roland et al., 2021; Wittenberg et al., 2002).

The main potential advantages of social insurance LTC schemes over tax-funded schemes comprise greater clarity, fairness and acceptability. Greater clarity arises not only because of the (at least perceived) link between contributions and benefits but also because countries with social insurance systems may have eligibility criteria that are more detailed and specific than countries with tax-based systems (Barber et al., 2021; Roland et al., 2021; Wittenberg et al., 2002). Greater fairness arises because social insurance contributions are usually based on earnings, which means that they are progressive, so long as there is no upper earnings limit on liability to pay contributions, while resources for tax-based schemes are generally raised through a combination of direct taxes such as incomes taxes which are progressive and indirect taxes which are regressive. Greater acceptability may arise from the hypothecation of social insurance contributions for LTC. The public may be more willing to pay higher contributions ear-marked for care than higher taxes which could potentially be used for care (Barber et al., 2021; Roland et al., 2021; Wittenberg et al., 2002). This may explain why the UK government has introduced a supplement to local council taxes hypothecated for social care and will shortly introduce a new levy hypothecated for health and social care.
